# *Cytisus scoparius* and *Ulex europaeus* Produce Volatile Organic Compounds with Powerful Synergistic Herbicidal Effects

**DOI:** 10.3390/molecules24244539

**Published:** 2019-12-11

**Authors:** María Pardo-Muras, Carolina G. Puig, Nuria Pedrol

**Affiliations:** 1 Department of Plant Biology and Soil Science, Faculty of Biology, University of Vigo, 36310 Vigo, Spain; mpardomuras@uvigo.es (M.P.-M.); cgpuig@uvigo.es (C.G.P.); 2 CITACA, Agri-Food Research and Transfer Cluster, Campus da Auga, University of Vigo, 32004-Ourense, Spain

**Keywords:** allelochemicals, gorse, phytotoxicity, reversibility, Scotch broom, synergism, weed control

## Abstract

New herbicides based on natural products are claimed to address weed resistance and environmental concerns related to synthetic herbicides. In our previous studies, certain volatile organic compounds (VOCs) produced by *Ulex europaeus* and *Cytisus scoparius* were argued to be responsible for the phytotoxicity of both shrub species. Interactions among VOCs were hypothesized to explain the inconsistency between the effects of the identified pure compounds and those naturally emitted from fresh plant material. In this work, eugenol, verbenone, terpinen-4-ol, *α*-terpineol, and linalool were assayed as binary mixtures of *Amaranthus retroflexus* and *Digitaria sanguinalis*. Powerful synergistic inhibitory effects were revealed for germination and early growth. Only 3.1 ppm of verbenone was enough to inhibit *A. retroflexus* germination when paired to other VOCs. Eugenol was capable of exacerbating the effects of terpinen-4-ol on *A. retroflexus*, even though it was innocuous when acting alone at 12.5 ppm. The verbenone and linalool pair produced very significant synergistic effects in terms of *D. sanguinalis* germination. The synergistic effects were predominantly irreversible for *D. sanguinalis*, since seeds exposed to paired VOCs were unable to recover their germination capacity after removing the phytotoxins or produced damaged seedlings. Both shrub species have been revealed as sources of natural herbicide molecules, with promising synergistic modes of action that deserve to be studied in depth.

## 1. Introduction

Since the first implementation of synthetic herbicides in crop protection systems, weeds have incessantly been developing resistances [1]. The main reason for such fast evolution is the long-term exploitation of synthetic herbicides with only one molecular target site. Commercial herbicides sum up to no more than 20 mechanisms of action (MOAs) [2] and are often poorly defined. Surprisingly, since the 1980s, no new herbicides with new MOAs have been introduced [3,4]. As a consequence, to date, there have been 505 herbicide-resistant weed biotypes described in 70 countries. From them, Australia, Brazil, Canada, China, France, Spain, and the USA have contributed to 50% of the registered herbicide resistance cases [5]. The linear increase of resistant weeds, coupled with the lack of new MOAs, threatens to render all existing herbicides unusable by 2050 [3]. Moreover, the inappropriate application of present herbicides has contributed to the increase in environmental pollution, besides producing toxicological problems in health [6]. These issues have become a major challenge for many agricultural producers.

Consequently, one of the primary objectives of weed research in recent years has been the search for natural compounds with new MOAs and target sites that have not been previously exploited. Plant secondary metabolites with recognized ecological functions (i.e., allelochemicals) offer an unparalleled source of structural diversity, with little MOA overlap with existing herbicides [1,7]. Especially, volatile organic compounds (VOCs) have aroused great interest as natural herbicides [8,9]. Also, plant secondary metabolites are putatively more environmentally friendly and rapidly degrade [1,10,11]. However, in nature, the phytotoxicity observed for many physiological processes is argued to never be limited to only one compound, but rather to mixtures, and often complex combinations of these molecules [12,13,14]. Thus, the interactions among allelochemicals deserve deep exploration, since compounds of different chemical classes and MOAs could be combined at lower individual concentrations in order to increase herbicide efficiency and minimize the development of herbicide resistance.

**Figure 1:**
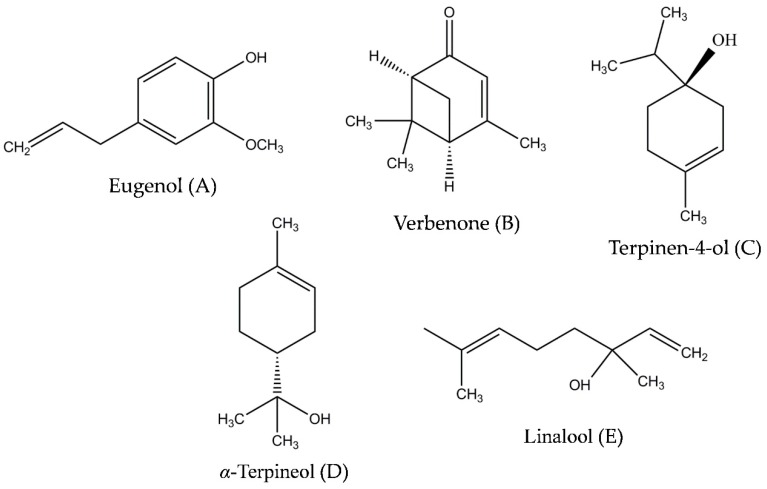
Structures of the volatile organic compounds used in this study: eugenol (**A**), verbenone (**B**), terpinen-4-ol (**C**), *α*-terpineol (**D**), and linalool (**E**).

A novel bioinspired strategy to combine a cocktail of bioactive compounds at low individual concentrations is the use of biomass of allelopathic species collected from the agroecosystem, which is incorporated into the soil as a bioherbicide [15,16,17]. Promising candidates are the abundant *Ulex europaeus* L. (gorse) and *Cytisus scoparius* (L.) Link. (Scotch broom). These two species are native to the Atlantic region and grow together in Atlantic shrubland [18]. They are considered highly invasive weeds outside of their natural distribution range [19,20]. Both species have often been reported for their bioactivity. Scotch broom extracts have shown antifungal [21], antimicrobial [22], and antioxidant activities [21,23], whereas gorse has been studied for its antioxidant [24] and antifungal [25,26] properties. However, their bioherbicide potential was first reported by Pardo-Muras et al. [27], who demonstrated that both species produce and emit VOCs capable of inhibiting the germination and early growth of the agricultural weed species *Amaranthus retroflexus* L. (redroot pigweed) and *Digitaria sanguinalis* (L.) Scop. (large crabgrass), two of the most problematic agricultural weeds in European crop production [28]. These authors described, for the first time, the presence of certain VOCs (Figure 1) with reputed bioherbicide effects in volatile extracts (determined by GC/MS) for both species, i.e., benzenoid eugenol (Figure 1A) in *U. europaeus*, and the oxygenated monoterpenes terpinen-4-ol (Figure 1C), *α*-terpineol (Figure 1D), and linalool (Figure 1E) among others, in *C. scoparius*. Based on dose-response bioassays with pure compounds, these and other VOCs have been argued to be involved in the phytotoxicity observed for the plant materials.

Moreover, verbenone (Figure 1B), another oxygenated monoterpene, identified in *C. scoparius*, was highlighted for its strong herbicidal activity, even at very low concentrations. Nevertheless, the concentrations of each of these allelochemicals in volatile extracts were far below the inhibition concentrations of the pure compounds assayed one to one. This finding led us to argue that the phytotoxicity of the plant materials might be due to the combined action of several compounds rather than due to individual activities. Thus, it is time to address the interactions of these natural compounds and to investigate their joint bioactivity.

In this work, the effects of binary mixtures of VOCs present in *U. europaeus* and *C. scoparius* are investigated in comparison with the phytotoxic activity of separate compounds, examining their effect on the germination and early growth of the agricultural weeds *A. retroflexus* and *D. sanguinalis*, similar to the reversibility of the phytotoxic effects observed in germination.

## 2. Results

### 2.1. Phytotoxicity of the Volatile Compounds Applied in Pairs

**Figure 2:**
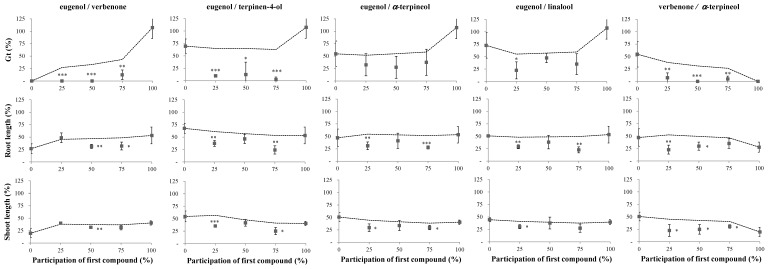

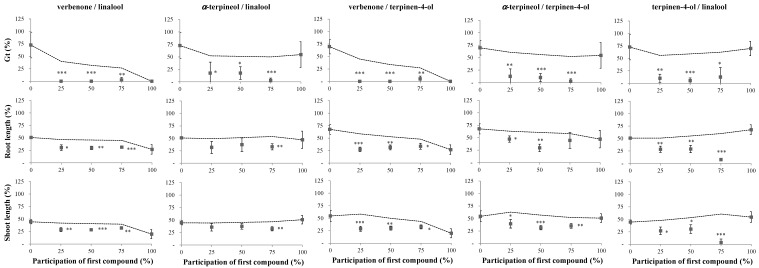
Effects of pairs of volatile compounds found in *Ulex europaeus* and *Cytisus scoparius* aerial biomass, assayed at fixed ratios, on the germination and early growth of *Amaranthus retroflexus*, expressed as percentages relative to the control (%). Values denote mean ± SD. The x-axis indicates the increasing participation of the first compound in the mixture (in percentage), i.e., 0, 3.1, 6.25, 9.4, and 12.5 ppm in the assayed volume. Dashed lines represent the expected effect of the two compounds acting independently [29]. Asterisks denote significant differences between observed and predicted values at * *p* ≤ 0.05; ** *p* ≤ 0.01; *** *p* ≤ 0.001; otherwise, not significant (*p* > 0.05).

**Figure 3:**
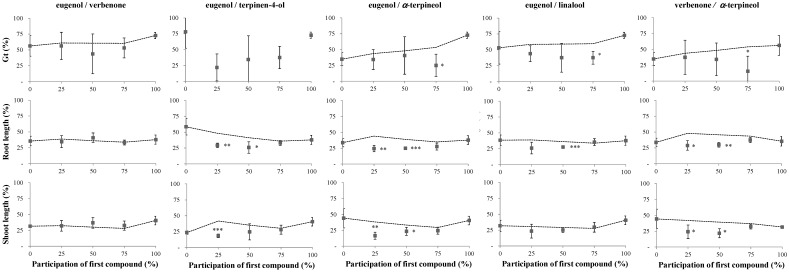

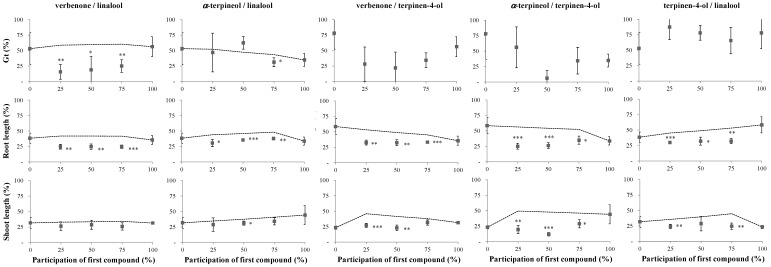
Effects of pairs of volatile compounds found in *Ulex europaeus* and *Cytisus scoparius* aerial biomass, assayed at fixed ratios, on the germination and early growth of *Digitaria sanguinalis*, expressed as percentages relative to the control (%). Values denote mean ± SD. The x-axis indicates the increasing participation of the first compound in the mixture (in percentage), i.e., 0, 3.1, 6.25, 9.4, and 12.5 ppm in the assayed volume. Dashed lines represent the expected effect of the two compounds acting independently [29]. Asterisks denote significant differences between observed and predicted values at * *p* ≤ 0.05; ** *p* ≤ 0.01; *** *p* ≤ 0.001; otherwise, not significant (*p* > 0.05).

Figure 2 and Figure 3 show the effects of the five most phytotoxic VOCs found in *U. europaeus* and *C. scoparius* aerial biomass, applied in pairs to the germination and early growth of *A. retroflexus* and *D. sanguinalis*. 

Except for two mixtures (eugenol/*α*-terpineol and eugenol/linalool, other than 75% linalool), all the assayed pairs at different proportions revealed statistically significant synergistic effects on the germination of *A. retroflexus*, with observed values below the values predicted by the model (Figure 2). Although 1 µL of eugenol, terpinen-4-ol, or linalool showed none or poor inhibition as isolated compounds, they revealed very intense synergistic effects on *A. retroflexus* germination, with reductions above 75% when combined at different proportions. The same compounds also increased the expected values of the highly phytotoxic verbenone when acting in a mixture, even attaining 100% inhibition of *A. retroflexus* germination.

In the case of the root and shoot lengths of *A. retroflexus*, significant deviations below the predicted values were observed for all the pairs tested at certain proportions. All the compounds applied alone attained 50% or more inhibition, but they were much more phytotoxic in paired combinations, attaining 75% growth inhibition in some cases. Despite the discrete reduction of root length by 1 µL terpinen-4-ol when acting individually, it showed significant to highly significant synergistic effects when combined with other compounds, thus achieving inhibitions from ca. 50 to 90% control (Figure 2). When 0.75 µL terpinen-4-ol was combined with 0.25 µL linalool, the volatile mixture almost completely inhibited the shoot and root growth of *A. retroflexus* (Figure 2).

From Figure 3, the results for *D. sanguinalis* germination showed far fewer cases of synergistic activity than the results for growth did. It was not possible to represent the predicted model for the germination of *D. sanguinalis* in the mixtures that included terpinen-4-ol, given its low phytotoxicity when acting alone (IC_50_ out of range, [27]). Otherwise, verbenone and linalool, when paired, revealed strong synergistic effects on *D. sanguinalis* germination at all tested proportions, attaining 85 to 75% germination inhibition compared to the ca. 50% predicted by the model. Moreover, synergistic effects were observed for the pairs eugenol/*α*-terpineol, eugenol/linalool, verbenone/*α*-terpineol, and *α*-terpineol/linalool, however, only at a concentration of 75% participation of the first compound, whereas synergistic effects were not observed for the pair eugenol/verbenone.

For the growth parameters measured on *D. sanguinalis*, all pairs at certain concentrations revealed significant deviations (*p* ≤ 0.05) below the predicted values, except for root length by eugenol/verbenone or shoot length by eugenol/verbenone, eugenol/linalool, and verbenone/linalool (Figure 3).

### 2.2. Reversibility Bioassays

VOCs in pairs were assayed for reversibility at concentrations that achieved a minimum number of ten non-germinated seeds. The total germination values (as a percentage relative to the control) of these pre-treated seeds, which were incubated in distilled water for 20 days, are represented in Table 1.

The seeds of *A. retroflexus*, which were pre-treated with different pairs of VOCs, experience 20 to 80% germination. The lowest values of reversibility were found for the pairs verbenone/*α*-terpineol and verbenone/terpinen-4-ol, at 75 and 25% participation of verbenone, respectively. Only verbenone acting alone revealed lower reversibility, with germination values of ca. 10%. For *D. sanguinalis*, the phytotoxic effects of the VOCs applied in pairs were much more persistent, showing germination values below 45% after being transferred to distilled water from all the proportions pre-assayed. Linalool and *α*-terpineol produced lower mean values of reversibility when acting individually than acting in the pairs with eugenol/linalool, verbenone/linalool, or *α*-terpineol/linalool, although the differences did not attain statistical significance. However, the pre-treated seeds that were capable of germinating after being transferred to distilled water resulted nevertheless in weed seedlings with yellowish radicles and an abnormal appearance.

## 3. Discussion

The phytotoxicity of certain volatiles potentially emitted by the flowering branches of gorse and Scotch broom have been previously demonstrated by Pardo-Muras et al. [27]. From the 20 and 28 VOCs identified in those species, respectively, the benzenoid eugenol and the oxygenated monoterpenes linalool, terpinen-4-ol, α-terpineol, and verbenone were argued to be the main VOCs responsible for such phytotoxicity, regarding their strong inhibitory effects on *A. retroflexus* and *D. sanguinalis*. It is noteworthy that verbenone was described as a powerful bioherbicide molecule, with IC_50_ and IC_80_ values (concentration required to obtain 50% or 80% inhibition of a given physiological parameter, respectively) for *A. retroflexus* germination of 3.69 and 7.39 ppm in the volume of the 9 cm diameter Petri dish, respectively, or 6.73 and 13.39 ppm for *D. sanguinalis* shoot length. Nonetheless, the concentration of verbenone (0.09 ppm) and other compounds such as linalool (0.14 ppm) or *α*-terpineol (0.06 ppm) in the plant volatile extracts were far lower than the ICs of the pure verbenone and other compounds separately assayed. To explain this apparent inconsistency, we hypothesized that the natural phytotoxicity of the shrub biomass could be due to the combined action of several compounds, rather than due to individual activities. In the present work, we shone light on this hypothesis by studying the binary interactions among some of these VOCs with reputed bioherbicide effects.

It is widely accepted that the phytotoxic effects observed in nature are due to additive, synergistic and/or antagonistic interactions among several natural compounds [12,13,14]. Nevertheless, to date, few studies have been reported which empirically support this argument, since reference works have dealt mostly with single compounds or natural extracts of poorly known composition, for which the interactive effects are only speculative. For the scarce amount of studies where the joint action of compounds has been tested [29,30,31,32], interactions have been based on the increase or decrease of the inhibitory activity relative to the effects of each substance alone, leading to the conclusion of the existence of synergism or antagonism, respectively. 

Within this frame of reference, we followed the model proposed by Vokou et al. [29], comparing the phytotoxic potential of the volatile compounds eugenol (found in the volatile extract of gorse), terpinen-4-ol, linalool, *α*-terpineol and verbenone (all of them found in the volatile extract of Scotch broom), acting as pairs against the seed germination and seedling growth of *A. retroflexus* and *D. sanguinalis*. All these VOCs have been reported to be phytotoxic against some target species, though not always with similar levels of bioactivity [27]. We have observed clear synergistic effects on the germination and/or early growth of both agricultural weeds for almost all of the pairs tested, though not with solely different putative MOAs, but also increased effects if compared to those of single compounds. This enhanced phytotoxicity of the paired mixtures (significantly greater than the model) means that these compounds act synergistically, and not only additively or summing their effects as when acting separately (this is close to the null model). Vokou et al. [29], found paired synergistic interactions between *α*-pinene/*β*-pinene, menthone/carvone, and geraniol/terpinen-4-ol, applied at different proportions, at a total volume of 2.5 µL, on the germination of *Lactuca sativa*, and between *α*-terpinene/*γ*-terpinene, (+)limonene/(−)limonene, and *ρ*-cymene/3-carene on the root elongation of the same target species. Previously, Asplund et al. [33] observed synergistic effects between the monoterpenes camphor, pulegone, and borneol, applied in pairs, on the germination of radish seeds. Likewise, Feng et al. [32] reported that sarmentosine exhibited synergistic effects against *Echinochloa crus-galli* and *A. retroflexus* when it was applied in combination with sarmentine. Here, we described some new highly significant synergistic effects of different pairs applied at a relatively lower total quantity (1 µL; i.e., 12.5 ppm in the volume of the Petri dish) on two agricultural weed species, separating their phytotoxic effects on germination or early growth. In our case, even the most phytotoxic compounds (verbenone and *α*-terpineol) increased their inhibitory potential on both target weeds and physiological processes when acting together. Nevertheless, their joint effects were less persistent on germination than those of pure verbenone when applied alone. 

Although some weed seeds could recover their germination capacity after removing the phytotoxin, the resulting seedlings were damaged. Thus, we can consider the effects on the embryon as permanent or at least persistent [34,35]. Eugenol was innocuous for *A. retroflexus* germination when applied at 12.5 ppm, however, surprisingly, quantities of eugenol below 12.5 ppm were capable of exacerbating the effects of verbenone and terpinen-4-ol. 

It is worth emphasizing that the benzenoid eugenol was only present in *U. europaeus* vegetative parts [27] but produced synergies with other compounds present in *C. scoparius*. Both shrub species grow together in the agroecosystem, so, under a practical point of view, the joint use of both shrub species as bioherbicides [15,16,17] could provide interspecific synergies among compounds, thus enhancing the bioherbicide potential of each species used separately. Such interspecific interactions of *U. europaeus* and *C. scoparius* should be also significant for the improved control of *D. sanguinalis* early growth. The interest of combining species for weed control was previously suggested by Sturchio et al. [36], who demonstrated that the mixture of essential oils of clove (*Syzygium aromaticum*) and rosemary (*Rosmarinus officinalis*) had synergistic activity. 

In contrast with our results, Vokou et al. [29] found antagonistic interactions when less bioactive compounds were present in the paired mixture, stating that “for an essential oil to be extremely active, it is necessary the participation of one or more active compounds are among its constituents.” This statement should be qualified, for instance, by the fact that α-terpineol, terpinen-4-ol, and linalool, which produced discrete effects when acting alone at 12.5 ppm, however, they produced ca. 90% to absolute germination inhibition when acting as pairs at quite lower concentrations on *A. retroflexus* seeds. Also, the inhibitory effects of the pair *α*-terpineol/linalool on *D. sanguinalis* germination were more persistent than those of the same compounds when acting alone.

The results contained in this work extend the interest of gorse and Scotch broom biomass even more, this time, as sources of natural herbicidal products with putatively different and synergistic MOAs. Nevertheless, the whole potential interactions among their allelochemicals, both volatile and water-soluble compounds, deserve to be studied in greater depth [37]. Their knowledge would inspire new eco-friendly herbicide formulations that combine lower concentrations of active principles with different target physiological processes, thus decreasing the risk of resistance development.

## 4. Materials and Methods 

### 4.1. Standard Compounds and Target Species

All the compounds tested had been previously identified from the volatile extract of flowering branches of *U. europaeus* and *C. scoparius* [27]. Pure volatile compounds were purchased from Sigma-Aldrich (St. Louis, MO, USA) and were used as received, without further purification.

Two agricultural weed species, *A. retroflexus* and *D. sanguinalis* from Herbiseed (Twyford, England, UK), were used as representative dicot and monocot target species, respectively. *Amaranthus retroflexus* seeds were previously synchronized by soaking in distilled water for 15 days at 4 °C and then air-dried, whereas *D. sanguinalis* seeds were placed under light for 56 days at 4 °C.

### 4.2. Phytotoxicity Bioassays of the Volatile Compounds Applied in Pairs

The phytotoxicities of the most effective VOCs tested in Pardo-Muras et al. [27] were evaluated when acting as pairs against the germination and seedling growth of *A. retroflexus* and *D. sanguinalis* and were compared with the effects of the compounds acting alone. The following 10 pairs, out of a set of 5 compounds, were examined: Eugenol/verbenone, eugenol/terpinen-4-ol, eugenol/*α*-terpineol, eugenol/linalool, verbenone/*α*-terpineol, verbenone/linalool, *α*-terpineol/linalool, verbenone/terpinen-4-ol, *α*-terpineol/terpinen-4-ol, and terpinen-4-ol/linalool. The pairs were tested at proportions 0:1, 1:3, 1:1, 3:1, and 1:0 on a total volume of 1 µL (corresponding to 12.5 ppm in the Petri dish volume), respectively, which means that the volume of the first compound in each mixture was 0, 0.25, 0.5, 0.75, and 1 µL, respectively (i.e., 0, 3.1, 6.25, 9.4, and 12.5 ppm in the assayed volume). 

For the germination bioassays, twenty-five seeds were placed in 9 cm diameter Petri dishes with filter paper moistened with 4 mL of distilled water. Inside the top lid of the plate, a filter paper strip was fixed, and the corresponding concentration of each compound was added to it with a micropipette. In such a way, the seeds were exposed to the compounds volatilized inside the dish volume [29]. The control treatments consisted of Petri dishes without any added compound. The Petri dishes were immediately closed and sealed with Parafilm and incubated in the dark at a constant temperature of 27 °C. This temperature was appropriate for achieving optimal germination and the early growth of both weed species, allowing common conditions for the release of VOCs in all bioassays. The number of germinated seeds (rupture of seed coats and the emergence of radicle ≥ 1 mm; [38]) was counted every 12 h for *A. retroflexu*s and every 24 h for *D. sanguinalis*, until no further seeds germinated in the control dishes. The total percentage of germinated seeds (Gt) and the coefficient of the rate of germination (CRG) were calculated after the procedures of Chiapusio et al. [39] and De Bertoldi et al. [40].

For early growth bioassays, fifteen pre-germinated seeds (radicle length between 1 and 3 mm [38]) were used under the same conditions as for germination bioassays. Root and shoot lengths of the pre-germinated seeds were measured after 48 h, and mean values per dish were expressed as a percentage of the respective control. For each treatment, concentration, and target species, four replicates were carried out.

The effect of each pair of compounds was tested following the model of Vokou et al. [29]. According to this model, the combined action of two compounds acting independently in a mixture can be explained by the following equation:

(1) $$y=\frac{1}{1+\left(\frac{a}{L_{a}}\right)+\;\left(\frac{b}{L_{b}}\right)}$$

where *y* is the value for germination, root or shoot length for a certain concentration of the two compounds ‘a’ and ‘b’, whereas L_a_ and L_b_ are the concentrations of each compound that would be required to inhibit the germination or the early growth 50% relative to the control (IC_50_). L_a_ and L_b_ were calculated according to the dose-response curves of every single compound tested alone [27] 

For the cases where a = 0 or b = 0, the parameters L_a_ and L_b_, respectively, were calculated using the next equations:

(2) $$L_{a}=\frac{X\cdot y_{a}}{1-y_{a}}$$

(3) $$L_{b}=\frac{X\cdot y_{b}}{1-y_{b}}$$

where X is the total concentration used in the bioassay (12.5 ppm), and y_a_ and y_b_ are the mean values of germination, root length, or shoot length of each compound acting alone. 

In their null model, Vokou et al. [29] assumed that, in each pair, the compounds act independently, so that “the weaker compound behaves as if it was a diluted version of the stronger compound and, conversely, the strong compound behaves like a concentrated version of the weaker one.” After comparing the observed effects with those predicted by the null model, any significant deviation of a given pair of compounds above or below the model value means the existence of synergistic or antagonistic effects, respectively.

### 4.3. Reversibility Bioassays

For each assayed pair described above, the viability of the pre-treated non-germinated seeds of *A. retroflexus* and *D. sanguinalis* were evaluated according to the procedure described by Pardo-Muras et al. [27]. Pairs of compounds that inhibited the germination of at least ten target seeds per replicate were selected. Non-germinated seeds were incubated in 6-well dishes, at a rate of 10 seeds per well, placed on a filter paper layer wetted with 750 µL of distilled water. For each species, ten non-pretreated seeds per well were used as the control treatment. The seeds were incubated as described for the previous bioassays, but the total percentage of germinated seeds (Gt) was obtained after 20 days of incubation.

### 4.4. Statistical Analysis

Replicated experiments were conducted in a completely randomized design. The data were expressed as a percentage relative to the control and were tested for normality by the Kolmogorov–Smirnov test and the homogeneity of variance was observed by Levene’s test. For the bioassays of the effects of volatile compounds acting in pairs, the observed values for each compound in a mixture were compared with the respective expected values predicted by the model of Vokou et al. [29], using the t-test for paired samples. These comparisons were performed for each compound, ratio, target species, and assay. For the reversibility bioassays, the data were analyzed using a one-way ANOVA or independent samples Student’s t-test at *p* ≤ 0.05. Fisher’s Least Significant Difference test (LSD) (*p* = 0.05) was used for post hoc multiple comparisons.

Statistical analyses were carried out using the SPSS v.19 (IBM SPSS Inc., Chicago, IL, USA) software package for Windows. 

## 5. Conclusions

The phytotoxic effects of *Ulex europaeus* and *Cytisus scoparius* volatile extracts found in Pardo-Muras et al. [27] are probably derived from complex interactions among allelochemicals present at very low concentrations, rather than the individual activity of compounds. Besides, the paired joint action of volatile compounds, which is mainly synergistic, reduces the threshold concentration needed to cause the described phytotoxic effects. Both legume shrubs have been revealed as interesting sources of natural herbicide molecules, with potentially novel synergistic modes of action that deserve to in depth study.

## Figures and Tables

**Table 1:** Reversibility of the phytotoxic effects on the germination of two agricultural weeds species pre-treated with pairs of volatile compounds and then transferred to water in the absence of volatile organic compounds (VOCs). Data are expressed as percentages relative to the control ± standard deviation (SD). For each weed species, *p*-values of the effects of pairs were significant at *p ≤* 0.05, very significant at *p* ≤ 0.01, highly significant at *p* ≤ 0.001, and not significant at *p* ≤ 0.05 (determined by ANOVA or independent sample *t*-tests). For each weed species and pair, mean values labeled with distinct letters were significantly different at *p* ≤ 0.05 (Fisher’s Least Significant Difference test, LSD) for post hoc multiple comparisons). Percentages indicate the increasing participation of the first compound in the mixture, i.e., 0, 3.1, 6.25, 9.4, and 12.5 ppm in the assayed volume.

Pair	Pre-Treatment Participation of the First Compound (%)	Germination (% ± SD)					
		*Amaranthus retroflexus*		*p-*value	*Digitaria sanguinalis*		*p-*value
Eugenol/verbenone	0	10.0 ± 8.17	b	0.022	25.0 ± 19.1	a	0.412
	25	45.0 ± 10.0	a		45.0 ± 34.2	a	
	50	50.0 ± 25.8	a		20.0 ± 16.3	a	
	75	40.0 ± 16.3	a		35.0 ± 10.0	a	
	100	#			#		
Eugenol/terpinen-4-ol	0	#		0.493	#		0.064
	25	55.0 ± 19.1			5.0 ± 10.0	a	
	50	70.0 ± 34.6			30.0 ± 11.5	a	
	75	50.0 ± 11.5			20.0 ± 16.3	a	
	100	#			#		
Eugenol/*α*-terpineol	0	50.0 ± 20.0	ab	0.022	15.0 ± 10.0	a	0.538
	25	30.0 ± 25.8	b		20.0 ± 16.3	a	
	50	80.0 ± 16.3	a		15.0 ± 19.1	a	
	75	40.0 ± 16.3	b		5.0 ± 10.0	a	
	100	#			#		
Eugenol/linalool	0	#		0.704	13.2 ± 5.3	a	0.888
	25	80.0 ± 28.3	a		15.0 ± 10.0	a	
	50	60.0 ± 43.2	a		15.0 ± 19.1	a	
	75	75.0 ± 30.0	a		20.0 ± 16.3	a	
	100	#			#		
Verbenone/*α*-terpineol	0	50.0 ± 20.0	a	0.009	15.0 ± 10.0	a	0.749
	25	50.0 ± 24.6	a		15.0 ± 10.0	a	
	50	50.0 ± 25.8	a		25.0 ± 25.2	a	
	75	20.0 ± 11.3	b		30.0 ± 25.8	a	
	100	10.0 ± 8.17	b		25.0 ± 19.1	a	
Verbenone/linalool	0	#		0.023	13.2 ± 5.3	a	0.315
	25	55.0 ± 10.0	a		20.0 ± 16.3	a	
	50	40.0 ± 16.3	ab		25.0 ± 10.0	a	
	75	40.0 ± 28.3	ab		40.0 ± 28.3	a	
	100	10.0 ± 8.17	b		25.0 ± 19.1	a	
*α*-Terpineol/linalool	0	#		0.714	13.2 ± 5.3	a	0.720
	25	45.0 ± 19.1	a		25.0 ± 10.0	a	
	50	65.0 ± 19.1	a		30.0 ± 11.5	a	
	75	50.0 ± 38.3	a		20.0 ± 40.0	a	
	100	50.0 ± 20.0	a		15.0 ± 10.0	a	
Verbenone/terpinen-4-ol	0	#		0.039	#		0.227
	25	25.0 ± 10.0	b		30.0 ± 25.8	a	
	50	45.0 ± 11.23	a		15.0 ± 10.0	a	
	75	55.0 ± 14.3	a		45.0 ± 19.1	a	
	100	10.0 ± 8.17	b		25.0 ± 19.1	a	
*α*-Terpineol/terpinen-4-ol	0	#		0.292	#		0.726
	25	50.0 ± 25.8	a		10.0 ± 11.5	a	
	50	70.0 ± 25.8	a		20.0 ± 16.3	a	
	75	35.0 ± 25.2	a		15.0 ± 10.0	a	
	100	50.0 ± 20.0	a		15.0 ± 10.0	a	
Terpinen-4-ol/linalool	0	#		0.943	13.2 ± 5.3	a	0.906
	25	45.0 ± 19.1	a		#		
	50	50.0 ± 25.8	a		15.0 ± 19.1	a	
	75	50.0 ± 25.8	a		#		
	100	#			#		

^#^ Concentrations for which the minimum number of ten non-germinated seeds for the reversibility bioassay was not achieved.

## References

[1] Soltys D., Krasuska U., Bogatek R., Gniazdowska A., Price A.J., Kelton J.A. (2013). Allelochemicals as Bioherbicides-Present and Perspectives. Herbicides-Current Research and Case Studies in Use.

[2] Duke S.O. (2012). Why have no new herbicide modes of action appeared in recent years?. Pest. Manag. Sci..

[3] Westwood J.H., Charudattan R., Duke S.O., Fennimore S.A., Marrone P., Slaughter D.C., Swanton C., Zollinger R. (2018). Weed Management in 2050: Perspectives on the Future of Weed Science. Weed Sci..

[4] Dayan F.E. (2019). Current Status and Future Prospects in Herbicide Discovery. Plants.

[5] Heap I. The International Survey of Herbicide Resistant Weeds. www.weedscience.com.

[6] Kudsk P., Streibig J.C. (2003). Herbicides–a two-edged sword. Weed Res..

[7] Dayan F.E., Duke S.O. (2014). Natural compounds as next generation herbicides. Plant Physiol..

[8] Benvenuti S., Cioni P.L., Flamini G., Pardossi A. (2017). Weeds for weed control: Asteraceae essential oils as natural herbicides. Weed Res..

[9] Brilli F., Loreto F., Baccelli I. (2019). Exploiting plant volatile organic compounds (VOCs) in agriculture to improve sustainable defense strategies and productivity of crops. Front. Plant Sci..

[10] Duke S.O., Romagni J.G., Dayan F.E. (2000). Natural products as sources for new mechanisms of herbicidal action. Crop Prot..

[11] Singh H.P., Batish D.R., Kohli R.K. (2003). Allelopathic interactions and allelochemicals: New possibilities for sustainable weed management. Crit Rev. Plant. Sci.

[12] Einhellig F.A., Inderjit, Dakshini K.M.M., Einhellig F.A. (1994). Allelopathy: Current status and future goals. Allelopathy: Organisms, Processes and Applications.

[13] Inderjit, Duke S.O. (2003). Ecophysiological aspects of allelopathy. Planta.

[14] Reigosa M., Gomes A.S., Ferreira A.G., Borghetti F. (2013). Allelopathic research in Brazil. Acta Bot. Brasilica.

[15] Xuan T.D., Shinkichi T., Khanh T.D., Chung I.M. (2005). Biological control of weeds and plant pathogens in paddy rice by exploiting plant allelopathy: An overview. Crop Prot..

[16] Puig C.G., Gonçalves R.F., Valentão P., Andrade P.B., Reigosa M.J., Pedrol N. (2018). The consistency between phytotoxic effects and the dynamics of allelochemicals release from *Eucalyptus globulus* leaves used as bioherbicide green manure. J. Chem. Ecol..

[17] Puig C.G., Revilla P., Barreal M.E., Reigosa M.J., Pedrol N. (2019). On the suitability of *Eucalyptus globulus* green manure for field weed control. Crop Prot..

[18] Basanta M., Diaz Vizcaino E., Casal M., Morey M. (1989). Diversity measurements in shrubland communities of Galicia (NW Spain). Plant Ecol..

[19] Clements D.R., Peterson D.J., Prasad R. (2001). The biology of Canadian weeds. 112. Ulex europaeus L. Can. J. Plant Sci..

[20] Peterson D.J., Prasad R. (1998). The biology of Canadian weeds. 109. *Cytisus scoparius* (L.) Link. Can. J. Plant Sci..

[21] Sundararajan R., Koduru R. (2014). *Cytisus scoparius*: A review of ethnomedical, phytochemical and pharmacological information. Indo Am. J. Pharm. Res..

[22] Lores M., Pájaro M., Álvarez-Casas M., Domínguez J., García-Jares C. (2015). Use of ethyl lactate to extract bioactive compounds from *Cytisus scoparius*: Comparison of pressurized liquid extraction and medium scale ambient temperature systems. Talanta.

[23] Pinela J., Barros L., Carvalho A.M., Ferreira I.C. (2011). Influence of the drying method in the antioxidant potential and chemical composition of four shrubby flowering plants from the tribe Genisteae (Fabaceae). Food Chem. Toxicol..

[24] López-Hortas L., Conde E., Falqué E., Domínguez H. (2016). Flowers of *Ulex europaeus* L.–Comparing two extraction techniques (MHG and distillation). C. R. Chimi..

[25] Máximo P., Lourenço A., Feio S.S., Roseiro J.C. (2002). Flavonoids from *Ulex airensis* and *Ulex europaeus ssp. europaeus*. J. Nat. Prod..

[26] Spínola V., Llorent-Martínez E.J., Gouveia-Figueira S., Castilho P.C. (2016). *Ulex europaeus*: From noxious weed to source of valuable isoflavones and flavanones. Ind. Crops Prod..

[27] Pardo-Muras M., Puig C.G., López-Nogueira A., Cavaleiro C., Pedrol N. (2018). On the bioherbicide potential of *Ulex europaeus* and *Cytisus scoparius*: Profiles of volatiles organic compounds and their phytotoxic effects. PLoS ONE.

[28] Meissle M., Mouron P., Musa T., Bigler F., Pons X., Vasileiadis V.P., Otto S., Antichi D., Kiss J., Pálinkás Z. (2010). Pests, pesticide use and alternative options in European maize production: Current status and future prospects. J. Appl. Entomol..

[29] Vokou D., Douvli P., Blionis G.J., Halley J.M. (2003). Effects of monoterpenoids, acting alone or in pairs, on seed germination and subsequent seedling growth. J. Chem. Ecol..

[30] Araniti F., Graña E., Reigosa M.J., Sanchez-Moreiras A.M., Abenavoli M.R. (2013). Individual and joint activity of terpenoids, isolated from *Calamintha nepeta* extract, on *Arabidopsis thaliana*. Nat. Prod. Res..

[31] Silva E.A.D., Lobo L.T., Silva G.A., Souza Filho A.P.D.S., Silva M.N., Arruda A.C., Guilhon G.M.S.P., Santos L.S., Arruda M.S.P. (2013). Flavonoids from leaves of *Derris urucu*: Assessment of potential effects on seed germination and development of weeds. An. Acad. Bras. Cienc..

[32] Feng G., Chen M., Ye H.C., Zhang Z.K., Li H., Chen L.L., Chen X.L., Yan C., Zhang J. (2019). Herbicidal activities of compounds isolated from the medicinal plant *Piper sarmentosum*. Ind. Crops Prod..

[33] Asplund R.O. (1969). Some quantitative aspects of the phytotoxicity of monoterpenes. Weed Sci..

[34] Dudai N., Poljakoff-Maybe A., Mayer A.M., Putievsky E., Lerner H.R. (1999). Essential oils as allelochemicals and their potential use as bioherbicides. J. Chem. Ecol..

[35] Sobrero M.C., Ronco A., Castillo G. (2004). Ensayo de toxicidad aguda con semillas de lechuga (Lactuca sativa L.). Ensayos toxicológicos y métodos de evaluación de calidad de aguas. Estandarización, intercalibración, resultados y aplicaciones.

[36] Sturchio E., Boccia P., Zanellato M., Meconi C., Donnarumma L., Mercurio G., Mecozzi M. (2016). Molecular and structural changes induced by essential oils treatments in *Vicia faba* roots detected by genotoxicity testing. J. Toxicol. Environ. Health, Part. A.

[37] Pardo-Muras M., Puig C.G., Souto C., Pedrol N. More about the bioherbicide potential of *Ulex europaeus* and *Cytisus scoparius*: Profiles of water-soluble compounds and their phytotoxic effects. J. Chem. Ecol..

[38] Mayer A.M., Poljakoff-Mayber A. (1963). The Germination of Seeds.

[39] Chiapusio G., Sanchez A.M., Reigosa M.J., Gonzalez L., Pellissier F. (1997). Do germination indices adequately reflect allelochemical effects on the germination process?. J. Chem. Ecol..

[40] De Bertoldi C., De Leo M., Braca A., Ercoli L. (2009). Bioassay-guided isolation of allelochemicals from *Avena sativa* L.: Allelopathic potential of flavone C-glycosides. Chemoecology.

